# Effect of maternal excessive iodine intake on neurodevelopment and cognitive function in rat offspring

**DOI:** 10.1186/1471-2202-13-121

**Published:** 2012-10-08

**Authors:** Le Zhang, Weiping Teng, Yuhui Liu, Jing Li, Jinyuan Mao, Chenling Fan, Hong Wang, Hongmei Zhang, Zhongyan Shan

**Affiliations:** 1 Department of Endocrinology and Metabolism, First Affiliated Hospital of China Medical University, Shenyang 110001, People’s Republic of China; 2 Endocrinology Institute of China Medical University, Liaoning Provincial Key Laboratory of Endocrine Diseases, Shenyang 110001, People’s Republic of China

**Keywords:** Iodine deficiency, Iodine excess, Thyroid hormone, Hippocampus, Neurodevelopment

## Abstract

**Background:**

Iodine deficiency and iodine excess are both associated with adverse health consequences. Iodine deficiency during pregnancy leads to insufficient maternal thyroid hormone, subsequently causing irreversible adverse effects on the neurological and cognitive functions of the offspring. The results of our previous epidemiological study suggested that mild iodine excess might increase the prevalence of subclinical hypothyroidism. In the present study, female Wistar rats maintained on low-iodine grain were randomly assigned to three groups based on iodated water concentration: low iodine (LI, 1.2 μg/d), normal iodine (NI, 5–6 μg/d), and 3-fold high iodine (3HI, 15–16 μg/d). The present study investigated whether higher-than-normal iodine intake (3HI) by rats from before pregnancy until breastfeeding affects the postnatal (PN) neurodevelopment (PN7 and PN45) of their offspring during particularly sensitive periods in brain development.

**Results:**

After 12 weeks of treatment (before pregnancy), iodine concentrations in urine and thyroid tissue and circulating thyroxine of adult females correlated with iodine intake. Brain-derived neurotrophic factor (BDNF) expression in the hippocampi of pups on PN7 and PN45 was decreased in 3HI group compared to the NI controls (*P <* 0.05, all) On PN7 and PN45, the BDNF levels of the 3HI pups were 83.5% and 88.8%, respectively, that of the NI pups. In addition, the 3HI group had a higher neuroendocrine-specific protein A (NSP-A) level than the NI controls on PN7 (*P <* 0.05). NSP-A levels of the 3HI pups were 117.0% that of the NI pups. No significant difference was observed in the expressions of c-Fos or c-Jun in the hippocampal CA1 region of the 3HI group compared to the controls (*P >* 0.05). Results from the Morris water maze test revealed that pups of the 3HI group had mild learning and spatial memory deficits.

**Conclusions:**

The neurodevelopmental and cognitive deficits of the 3HI pups were mild and temporary, likely related to the changes in hippocampal protein expressions of BDNF and NSP-A.

## Background

Iodine is essential for the synthesis of thyroid hormone, including during pregnancy. Maternal thyroid hormones have essential roles in foetal brain development, regulating both morphological and biochemical changes before the onset of foetal thyroid function [1,2]. Moderate and severe iodine deficiency during pregnancy leads to insufficient maternal thyroid hormone, subsequently causing irreversible adverse effects on the neurological and cognitive functions of the offspring [3-5]. In China the Universal Salt Iodization (USI) policy was implemented in 1996 and since then the average resident’s iodine status was in excess for three years and more than adequate for six years. Previously, we conducted a five-year follow-up study from 1999 to 2004 to evaluate the effect of different iodine intake on thyroid diseases. We found that more than adequate (median urinary iodine [MUI] 243 μg/L) and iodine excess (MUI 651 μg/L) could increase the prevalence of subclinical hypothyroidism [6]. However whether more than adequate and excessive iodine intake can affect the neurodevelopment of the offspring has not been investigated.

The genes c-Fos and c-Jun are essential for spatial learning and memory consolidation in rats, and hypothyroidism due to iodine deficiency is associated with reduced expressions of c-Fos and c-Jun in the rat hippocampal CA1 region [7-9]. Maternal hypothyroidism affects the expression of foetal and neonatal brain-derived neurotrophic factor (BDNF) [10,11] and neuroendocrine-specific protein (NSP)-A [12], both of which are important mediators of thyroid hormone and have essential roles in brain development.

In our present study, female Wistar rats were maintained on low-iodine grain and randomly assigned to three groups based on the iodated water concentration: low iodine (LI), normal iodine (NI), and 3-fold high iodine (3HI) groups. Furthermore we explored whether maternal iodine excess in rats could lead to changes in neurological function in offspring.

## Results

### Urinary and thyroidal iodine content

Urine iodine concentrations of the adult female rats paralleled their iodine intake; urine and tissue iodine concentrations increased gradually with increasing iodine intake. In the low iodine (LI) group, urinary iodine was only 20.27% that of the normal iodine (NI) group, and the thyroidal iodine was 15.37% that of the NI group. At the same time, the 3-fold iodine (3HI) group had significantly elevated concentrations of iodine in urine and thyroid tissue, which was 307.16% and 141.92%, respectively, that of the NI group (*P <* 0.05, all; Table 1).

**Table 1:** Iodine concentration in thyroid tissue and urine of pre-pregnant females after 12 weeks of treatment

	**Iodine concentration (μg/L)**	
	**Urine**	**Thyroid tissue**
LI	37.48 ± 25.89 *	77.78 ± 33.19 *
NI	184.90 ± 23.27	506.05 ± 39.62
3HI	567.93 ± 69.84 *	718.20 ± 33.19 *

Values are expressed as the mean ± SEM (n = 6 for each group). *P   < 0.05 compared with normal iodine control group.

### Maternal and pup thyroid hormone

Maternal thyroid hormone: After 12 weeks of treatment (pre-pregnancy), total thyroxine (TT_4_) and free thyroxine (FT_4_) concentrations in the sera of the adult females were significantly lower in the LI group compared with those in the NI (*P* < 0.05, all). TT_4_ and FT_4_ levels in the maternal serum were significantly higher in the 3HI group than those in NI control group (*P <* 0.05, all). On gestational (G) day 17, TT_4_ and FT_4_ concentrations in sera of the adult females remained significantly lower in the LI group compared with those in the NI (*P* < 0.05, all), while the 3HI group still exhibited significantly higher circulating TT_4_ level compared with the control group (*P <* 0.05). However, on G17 the level of FT_4_ was a increasing trend, however no significant difference in FT_4_ concentration between the NI and 3HI groups. After 12 weeks of treatment (pre-pregnancy) and on G17, thyroid stimulating hormone (TSH) concentrations were significantly higher in the LI group compared with the NI (*P* < 0.05, all), while there were descending trends, although no significant changes were found in the TSH levels after 12 weeks of treatment (pre-pregnancy) and on G17 in the 3HI rats compared with these levels in the NI (*P* > 0.05; Table 2).

**Table 2:** Maternal thyroid hormone levels of the three treatment groups during pre-pregnancy and G17

	**12 weeks of treatment (pre-pregnancy)**			**G17**		
	**TSH (mIU/L)**	**TT**_**4**_**(μg/dL)**	**FT**_**4**_**(pmol/L)**	**TSH (mIU/L)**	**TT**_**4**_**(μg/dL)**	**FT**_**4**_**(pmol/L)**
LI	1.081 ± 0.27 *	1.15 ± 0.15 *	11.30 ± 0.70 *	0.120 ± 0.04 *	1.0 ± 0 *^, a^	9.51 ± 0.31 *
NI	0.063 ± 0.02	3.73 ± 0.41	25.10 ± 0.89	0.034 ± .000	2.66 ± 0.41	24.30 ± 2.77
3HI	0.029 ± 0.01	5.52 ± 0.95 *	29.23 ± 0.62 *	0.023 ± 0.01	3.95 ± 0.38 *	27.00 ± 3.34

Values are expressed as mean ± SEM; n = 6 for each group. *P < 0.05 compared with the NI control on the same day; ^a^The sensitivity of the chemiluminescence immunoassay for TT_4_ was 1.0 μg/dL.

Pup thyroid hormone: On postnatal (PN) day 7 (PN7) and day 45 (PN45), the sera of pups in the LI group had significantly higher TSH levels, and lower TT_4_ and FT_4_ levels, than those in the NI group (*P <* 0.05, all). On PN7, pups from the 3HI group had a significantly higher serum TSH level compared with NI pups (*P <* 0.05). However, there was no significant difference between the 3HI and NI groups in TT_4_ and FT_4_ levels. On PN45 there was no significant difference in any of the serum thyroid hormone levels between pups of the 3HI and NI groups (*P >* 0.05, all; Table 3). This indicated that the thyroid dysfunction of the pups from the 3HI group could be rectified by PN45.

**Table 3:** Hormone levels in offspring of the three treatment groups on PN7 and PN45

	**PN7**			**PN45**		
	**TSH (mIU/L)**	**TT**_**4**_**(μg/dL)**	**FT**_**4**_**(pmol/L)**	**TSH (mIU/L)**	**TT**_**4**_**(μg/dL)**	**FT**_**4**_**(pmol/L)**
LI	0.157 ± 0.03 *	1.48 ± 0.99 *	7.71 ± 0.24 *	0.320 ± 0.06 *	2.84 ± 0.32 *	23.62 ± 1.25*
NI	0.079 ± 0.01	2.44 ± 0.10	10.35 ± 0.34	0.061 ± 0.01	4.86 ± 0.61	34.68 ± 3.01
3HI	0.142 ± 0.02 *	2.45 ± 0.18	9.69 ± 0.32	0.080 ± 0.02	5.03 ± 0.53	33.80 ± 1.87

Values are expressed as mean ± SEM; n = 6 for each group. *P < 0.05 compared with NI control on the same day.

### Effect of 3HI on the protein expressions of c–Fos and c-Jun in the CA1 area of hippocampus

**Figure 1:**
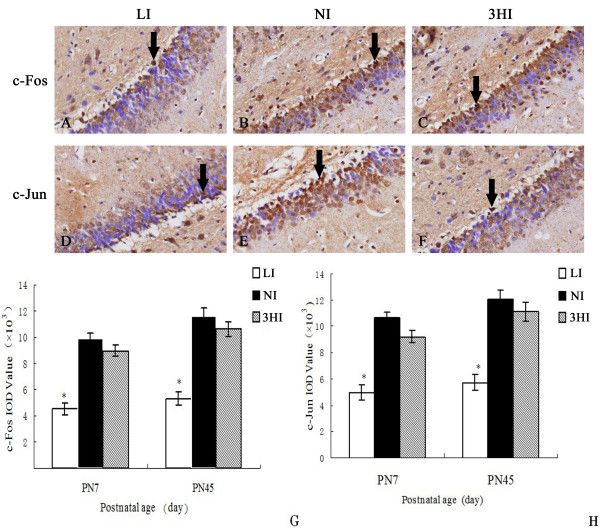
**Expression of c-Fos and c-Jun in the hippocampal CA1 area of pups on PN7 and PN45. **Upper three photomicrographs (**A**-**C**) show the expression of c-Fos in the CA1 of the LI (A), NI (B), and 3HI (C) groups. Lower three photomicrographs (**D**-**F**) show the expression of c-Jun in the CA1 of the LI (D), NI (E), and 3HI (F) groups. **G** and **H** represent the IOD values of c-Fos and c-Jun expressions in CA1 on PN7 and PN45, respectively. Data are expressed as the mean ± SEM (n = 6, for each group). * *P <* 0.05 compared with the NI control group on the same day.

Photomicrographs of the immunohistochemistry-stained hippocampal tissues from PN7 pups showed positive expression of c-Fos and c-Jun in area CA1 in all treatment groups (Figure 1A-F). The IOD values of c-Fos and c-Jun in CA1 area were significantly decreased in the pups of the LI group compared with control pups (*P <* 0.05). However, there was no significant difference in c-Fos and c-Jun expressions between the 3HI and NI groups (*P >* 0.05). On PN45, the expression of c-Fos and c-Jun in the LI group was also the lowest among the three groups (*P <* 0.05). There was no significant difference in the expressions of c-Fos and c-Jun in CA1 region of hippocampus between the 3HI pups and dams of the NI group (*P >* 0.05).

### Effect of 3HI on BDNF and NSP-A expression in the hippocampus

**Figure 2:**
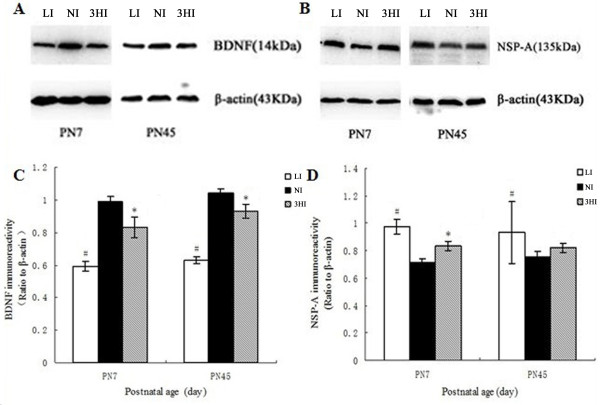
**Protein expression levels of BDNF and NSP-A in the pup hippocampus on PN7 and PN45. A** and **B**: protein levels determined via Western blot. **C** and **D**: Ratios of NSP-A/β-actin and BDNF/β-actin immunoreactive densities were determined for each group. The expression of the two proteins was normalized to β-actin. The height of each bar represents the mean ± SEM (n = 6 for each group). * *P <* 0.05 compared with the NI group on the same day; ^#^*P <* 0.01 compared with the NI group on the same day.

On PN7 and PN45, BDNF protein expression levels were significantly lower in both the LI and 3HI pups compared with the NI controls. Specifically, on PN7 the BDNF levels in the pups of the LI group were 58.84% that of the NI (*P <* 0.01) , and the BDNF levels of the pups in the 3HI group were 83.5% that of the NI (*P <* 0.05). On PN45, the BDNF levels in the pups of the LI group were 59.78% that of the NI (*P <* 0.01), and the BDNF levels of the pups in the 3HI group were 88.8% that of the NI (*P <* 0.05; Figure 2A and C).

On the other hand, on PN7 the expression of NSP-A was higher in the LI and 3HI pups when compared with the NI controls: The levels of NSP-A of the LI and 3HI pups were 136.7% and 117.0%, respectively, that of the NI pups (*P <* 0.01 and *P <* 0.05, respectively). NSP-A protein expression levels were significantly higher in the LI pups compared with the NI controls on PN45. The levels of NSP-A of the LI pups were 123.6% that of the NI pups (*P <* 0.05). However, no significant differences in the NSP-A levels were observed between the 3HI dams and NI dams on PN45 (*P >* 0.05; Figure 2B and D).

### Morris water maze (MWM) test

**Figure 3:**
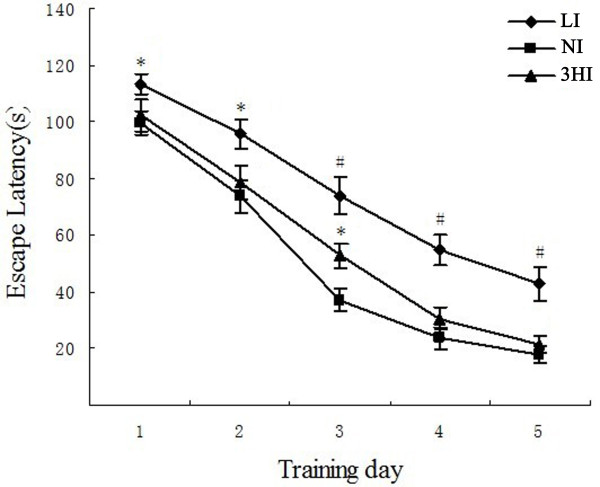
**Performance of pups in the Morris water maze. **Data are expressed as the mean ± SEM (n = 10 for each group). * *P <* 0.05 compared with the NI group on the same day; ^#^*P <* 0.01 compared with the NI group on the same day.

To evaluate whether various levels of iodine intake during gestation could cause cognitive and behavioral alterations in offspring, the pups underwent the MWM test on PN40-44. The time to reach the hidden platform (escape latency times) of all treatment groups became shorter as the number of training trials increased. However, the escape latencies were significantly longer for pups in the LI group compared with the NI group. Interestingly, our data showed that the 3HI pups took more time than the controls to learn the spatial cues required to find the hidden platform for all 5 days of testing, and there was a statistical difference when compared with the NI control group on the third day (*P <* 0.05; Figure 3).

## Discussion

Much recent attention has focused on elucidating the effects of iodine deficiency on neurodevelopmental impairment [13,14]. However, the effects of iodine excess should not be overlooked. We investigated whether and how a 3-fold increase in the physiological dose of iodine in rat would affect brain development of their offspring. The rat brain at PN7 is considered developmentally equivalent to the human brain at birth [15]. Rat brain development at PN21 approximates the late toddler stage of humans, and PN45 equates to the teenager stage [16]. We selected PN7 and PN45 as two crucial time points in the brain development of the rat to explore the effect of maternal excessive iodine intake on the neurodevelopment and cognitive function in their offspring. Our results show for the first time that there were changes in BDNF and NSP-A expression and neurodevelopmental impairment in rat pups whose mothers had a 3-fold higher-than-normal intake of iodine prior to pregnancy and throughout gestation and breastfeeding.

Iodine intake levels that are either lower or higher than the recommended range are associated with an increase in the incidence of thyroid disease in China [6]. Both thyroid hormone deficiency and excess have detrimental effects on the expression of neuronal proteins during neurodevelopmental stages [17,18].

Iodine deficiency is the most common cause of hypothyroidism. The results of some studies regarding excess iodine suggested that high iodine intake was associated with increased incidence of hypothyroidism [19,20], while others found that it led to hyperthyroidism [21,22]. These different results may be related to the duration of high iodine exposure. The incidence of hyperthyroidism after the initiation of the Universal Salt Iodization (USI) program began to rise at about 6 months, peaked at 1–3 years and returned to the baseline within 3–10 years [23-25]. Iodine-induced thyrotoxicosis lasting the first few years after the implementation of iodine supplementation was reported in Lesotho [26] and Poland [27]. However our epidemiological study demonstrated that there was no difference in either the prevalence or the incidence of hyperthyroidism among mildly deficient, more than adequate, and excessive iodine intake areas (MUI 84, 243, and 651 μg/L, respectively). We found that more than adequate and excessive iodine intake could increase the prevalence of subclinical hypothyroidism [6]. The USI policy had been implemented for 3 to 8 years in China, since 1996, when we conducted the epidemiological study from 1999 to 2004. Therefore we did not obtain epidemiological data for the early stage of iodine supplementation.

In the present animal study, after 12 weeks of treatment (pre-pregnancy), the serum TT_4_ and FT_4_ levels were significantly higher in the adult female rats of the 3HI group compared with those of the NI group. On G17, the TT_4_ concentration of the adult females remained the higher level in the 3HI group compared with that of the NI. Although no significant changes were found in the TSH levels on pre-pregnancy and G17 in the 3HI rats compared with those of the NI, there were descending trends in the 3HI group.

Before the onset of foetal thyroid function in both humans (10–12 weeks gestation) and rats (G17), early foetal brain development is completely dependent on maternal thyroxine (T_4_) supply [28]. Even after the foetal thyroid has begun to secrete hormone, as much as 17.5% of foetal T_4_ derives from transplacental transfer [29]. Recent studies have reported that excess thyroid hormone could impair foetal brain development and affect the neurological outcome of rat offspring [30,31]. Excessive iodine intake has been shown to inhibit foetal thyroid function and lead to iodine-induced neonatal hypothyroidism in Asian populations [32]. Our previous study [33] found that excessive iodine intake increased the risk of subclinical hypothyroidism in offspring. Furthermore, excessive iodine content from breast milk caused subclinical hypothyroidism in preterm infants [34]. These studies were consistent with our finding in the present study that maternal iodine excess caused subclinical hypothyroidism of rat pups on PN7, although the thyroid hormone levels of pups in the 3HI group were within the normal range on PN45. Our results indicated that maternal thyroid hormone disruption during sensitive periods of brain development caused by mildly maternal iodine excess could lead to thyroid dysfunction of pups and cause neurodevelopmental defects in offspring.

The proteins c-Fos and c-Jun in the nuclei of neurons are involved in neuroplastic mechanisms and neuronal differentiation, and also have important roles in memory formation and consolidation [8,35]. In our study, c-Fos and c-Jun expression in the hippocampal CA1 area of rat pups was not significantly affected by the 3-fold high iodine intake of their mothers during gestation. It is possible that compensatory mechanisms restored c-Fos and c-Jun to normal levels in spite of maternal thyroid dysfunction. Another possibility is that c-Fos and c-Jun were not involved in the neurodevelopmental impairment of pups from the 3HI group.

BDNF, a neurotrophin protein, has significant influence on crucial processes of brain development, including neurogenesis, neuronal differentiation, synaptogenesis, memory formation, and consolidation [36-39]. Previous reports have documented that maternal thyroid dysfunction could affect the expression of BDNF and cause neurological defects in neonates [40] and adult rats [11]. Our previous studies [10,41] reported that maternal subclinical hypothyroidism decreased BDNF expression in rat pup hippocampi and impaired spatial learning; pups required more time during the Morris water maze test to find the hidden platform, compared with pups from NI mothers. The present study found that BDNF protein levels were decreased in the hippocampi of the pups from both the 3HI and LI mothers. On PN45, the BDNF expression in the 3HI pups remained lower than control pups, although circulating levels of thyroid hormone had fully recovered. Our study indicated that a persistently lower BDNF level may contribute to the adverse effect of 3HI on the developing brain. However, the impairment was mild compared with hypothyroidism induced by iodine deficiency.

NSP-A is known to be an important mediator of thyroid hormone effects during brain development and is involved in neuronal differentiation and axonal guidance [42]. Dowling *et al.*[12,43] showed that the expression of NSP-A was regulated by thyroid hormone, and the expression of NSP-A was selectively affected by maternal hypothyroidism in the proliferative zone of the fetal rat brain cortex. Our data also found that the expression of NSP-A in the hippocampus on PN7 was affected by mildly maternal excessive iodine intake. The abnormal expression of NSP-A in the neonatal brain appears to be related to the neurodevelopmental impairment in the pups of the 3HI group, but the impairment was less severe than pups in the LI group.

In this study, spatial learning ability and memory of the rat pups were assessed using the MWM test on PN40-44. Pups in the LI group required more time to find the hidden platform, compared with pups from NI mothers. The severe impairment of spatial and learning ability was associated with the thyroid dysfunction and abnormal levels of proteins related to neurodevelopment, which were not reversed by PN45 in LI group, and the neurodevelopmental impairment thus appeared to be permanent. The mean escape latency of 3HI pups was longer than for the NI control pups, and the difference was significant on the third day (PN42; *P <* 0.05). Our results suggested that the offspring in the 3HI group may have had a mildly impaired learning capacity, which could be associated with a decrease in BDNF and an increase in NSP-A levels. The mild spatial learning and memory impairment was temporary, which was consistent with the recovery of NSP-A expression on PN45, and the long-term effects of mildly maternal excessive iodine intake on neurodevelopment and cognitive function need to be investigated in our future study.

## Conclusions

In summary, this study found that both low and high levels of iodine intake by rats could affect the neurological development of offspring. Reduced the expression of BDNF and enhanced that of NSP-A during hippocampal development of the offspring might be related with the impaired cognitive functions. However the impairment induced by maternal 3-fold high iodine intake was mild and temporary, which suggested that careful control of maternal iodine intake level is important to prevent neurodevelopmental defects in offspring.

## Methods

### Animals

Specific Pathogen-Free (SPF) female Wistar rats (n = 60) weighing 80–120 g were obtained from HFK Bioscience Laboratory Animal (Beijing, China). The Animal Research Committee of China Medical University approved this study. All experiments and procedures were carried out in accordance with the Guide for the Care and Use of Laboratory Animals mandated by the National Institutes of Health. Rats were housed under SPF conditions at 24 ± 2°C under automatic 12- h light and 12- h dark cycles.

Animals were randomly assigned to one of three treatment groups (n = 20, each): LI, NI, and 3HI. All groups were administered a low iodine diet (60 μg iodine per kg of feed, or 1.2 μg iodine in 20 g of feed per rat per day) of corn (73%), millet (20%), and soybean (7%) obtained from an area that is severely iodine-deficient (Hebei, China). Other chemical and trace elements were added, based on the standard American Institute of Nutrition (AIN)-93 diet. With the exception of iodine content, the rats’ food was nutritionally complete.

Rats in the LI group were watered with deionized water only and therefore received iodine only 1.2 μg/d, from their feed. Rats of the other groups were given potassium iodate (KIO_3_) dissolved in deionized water: the NI control group received 140 μg/L (5–6 μg/d), and the 3HI group 480 μg/L (15–16 μg/d). The rats were fed with the low iodine diet and administered with drinking water containing different concentrations of iodate from pre-pregnancy (12 weeks) until their pups reached PN21.

After 12 weeks of treatment, 6 female rats in each group were weighed and anesthetized with 10 % chloral hydrate. Serum and thyroid samples were obtained and stored. Remaining rats (n = 14, each group) were mated with normal male Wistar rats (female:male = 2:1) and the next day a vaginal smear was obtained and analysed under a microscope to confirm the presence of spermatozoa. The rats with a smear positive for spermatozoa were considered mated and the day was recorded as G0. On G17, blood samples were collected from each pregnant rat of all groups for serum hormone analysis.

The pups from the rat mothers of each treatment group (LI, NI, and 3HI) were permitted free access to normal food and water from PN21 until PN45. On PN7 and PN45, the brains of six pups from each group (equal numbers of male and female pups from each group were chosen) were rapidly removed for immunohistochemistry and Western blot. Blood samples were drawn from the heart of the pups and serum were stored at −70°C.

On PN40-44, a Morris water maze was used for evaluating spatial learning and memory in the remaining offspring.

**Figure 4:**
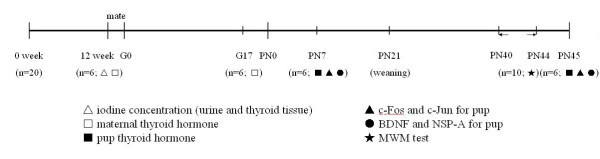
**Schematic of experimental timeline. **The rats were fed with the low iodine diet and administered with different drinking water from pre-pregnancy (12 weeks) until their pups reached PN21. LI (1.2 μg/d); NI (5–6 μg/d); 3HI (15–16 μg/d). The pups from the rat mothers of each treatment group (LI, NI, and 3HI) were permitted free access to normal food and water from PN21 until PN45. G, gestational day; PN, postnatal day; MWM, Morris water maze.

A schematic diagram of the experimental design and timeline is shown in Figure 4.

### Urinary and thyroidal iodine content measurement

The rats were fed with the low iodine diet and administered with different drinking water for 12 weeks. Then 6 rats were randomly taken from different groups and urine samples were collected within 24 hours before euthanization. The rats were then deeply anesthetized and thyroid tissue samples were obtained. The iodine concentration was determined in urine and thyroid tissue homogenate by arsenic cerium catalytic spectrophotometry.

### Hormone measurements

Blood samples were centrifuged at 3400 × *g* for 15 min. Serum TT_4_, FT_4_, and TSH concentrations were analysed via chemiluminescent immunoassay (IMMULITE, Diagnostic Products, CA). The limit of detection for TT_4_ was 1.0 μg/dL, and any sample below this level was recorded as 1.0 μg/dL for statistical purposes. The upper limit of detection of TT_4_ was 24.0 μg/dL. For FT_4_, the lower and upper limits of detection were 3.9 pmol/L and 77.2 pmol/L, respectively. For TSH, the lower and upper limits of detection were 0.002 mIU/L and 75 mIU/L, respectively.

### Immunohistochemistry

On PN7 and PN45, 6 pups were taken from different litters in each group, were deeply anesthetized and perfused with 200 mL saline followed by 200 mL 4% paraformaldehyde. Rat brains were embedded in paraffin and sectioned coronally with a microtome into 5 μm-sections. Sections were dewaxed and rehydrated and treated for endogenous peroxidase with 3% methanol-hydrogen peroxide for 10 min.

All sections were incubated with the primary antibodies (c-Fos, 1:2000; c-Jun, 1:400; Abcam Biochemicals, England) at 4°C overnight and were then incubated with serum for 10 min at room temperature. The tissue sections were incubated in biotin-conjugated secondary antibodies (Maixin, Fuzhou, China) for 30 min at 37°C, and in streptavidin-peroxidase complex (Maixin, Fuzhou, China) for 10 min. Sections were treated with a solution of 3, 3’-diaminobenzidine (DAB; Maixin, Fuzhou, China) for 3 to 10 min depending upon the staining of the DAB reaction product observed under light microscopy. Finally, the sections were counterstained with hematoxylin, dehydrated, rinsed, and mounted in neutral gum (China National Medicines, Shanghai, China).

The hippocampal CA1 area of all sections was observed at 400×. The integral optical density (IOD) values that indicated the expression levels of the proteins were measured using Image-Pro Plus 5.0 software (Media Cybernetics, Silver Spring, MD, USA).

### Western blot

On PN7 and PN45, the brains of the 6 pups per treatment group were perfused with 200 mL 0.9% saline. Hippocampal samples were collected to assay BDNF and NSP-A protein expression by Western blot. Tissue samples were washed in lysis buffer containing protease and phosphatase inhibitors (Keygen Biotech, Nanjing, China), homogenized by sonication, and centrifuged at 10,000 *× g* for 10 min at 4°C. The supernatants were collected and the protein concentration was measured via the Coomassie brilliant blue assay. Tissue lysates were diluted and then boiled at 100°C for 5 min. Samples (50 μg) were fractionated via sodium dodecyl sulfate polyacrylamide gel electrophoresis (10% gel for NSP-A, and 15% gel for BDNF). Proteins were transferred onto a nitrocellulose membrane and blocked with 5% skim milk in Tris-buffered saline containing 0.1% Tween-20 for one hour at room temperature. The membranes were washed and incubated with primary antibody (Rtn-1A [NSP-A] 1:500, Santa Cruz Biotechnology, USA; or BDNF 1:1000, Millipore, USA). An antibody against β-actin (1:1000; Santa Cruz Biotechnology, USA) was used as a reference. After incubation with primary antibodies, membranes were incubated with corresponding horseradish peroxidase-conjugated secondary antibodies (1:5000; Zhongshan Golden Bridge Biotechnology, China) before reaction with an enhanced chemiluminescence solution (Alphaview 1.3, USA). The films were scanned, and the protein band intensities were quantified with an image analysis program.

### Morris water maze test

The MWM was designed to assess spatial learning and memory in rodents [44]. The apparatus consists of a black circular swimming pool (120 cm diameter, 50 cm depth) that was filled with water (24 ± 2°C) mixed with black edible pigment. The pool’s four quadrants of equal area were designated 1, 2, 3, and 4. A circular platform (10 cm diameter) was located 2 cm below the water surface in the middle of quadrant 1. The platform was the same colour as the black swimming pool water so that it the rats could not see it. The two investigators who administered the water maze test were always in the same position in the room.

The MWM test was performed on PN40-44. The entire test took 5 consecutive days with 8 training trials per day and 30–60 s inter-trial periods between two consecutive trials. The rat pups from the three groups (n = 10, each) were moved to the procedure room 30 min before testing. In each trial the rat was placed into the water, immediately facing the wall of the pool at an obvious compass location. The time required for the rat to find the platform (escape latency time) was recorded, with a maximal time of 120 s allowed. If the rat failed to locate the platform in the allowed time, a latency of 120 s was recorded. The rat was guided manually to the platform and allowed to stay on it for 10 s before being returned to its home cage.

### Statistical analysis

All analyses were carried out using SPSS 17.0 software; the analyser was blind to the identity of the groups. All data results are presented as mean ± SEM. Differences among the three groups were analysed using one-way analysis of variance (ANOVA). When the F-value indicated significance, post-hoc test (LSD) was used to correct for multiple comparisons. Differences were considered statistically significant at *P <* 0.05.

## Abbreviations

3HI: 3-fold iodine; AIN: American Institute of Nutrition; ANOVA: Analysis of variance; BDNF: Brain-derived neurotrophic factor; FT_4_: Free thyroxine; G: Gestational LI: Low iodine; KIO_3_: Potassium iodate; MUI: Median urinary iodine; MWM: Morris water maze; NI: Normal iodine; NSP: Neuroendocrine-specific protein; PN: Postnatal; SPF: Specific pathogen-free; TSH: Thyrotropin or thyroid stimulating hormone; TT_4_: Total thyroxine; USI: Universal salt iodization.

## Competing interests

The authors declare that they have no competing interests.

## Authors’ contributions

LZ, WT, and ZS conceived of the study and participated in its design and coordination. LZ drafted the manuscript. LZ, YL, JM, CF, HW and HZ carried out the experiments, collected and analyzed the data. WT and ZS revised the manuscript. All authors participated in writing the manuscript. All authors read and approved the final manuscript.

## References

[B1] de Escobar GM Obregón MJ del Rey FE Role of thyroid hormone during early brain development Eur J Endocrinol 2004 151 Suppl 3 U25 U37 15554884 10.1530/eje.0.151u025

[B2] Zoeller RT Rovet J Timing of thyroid hormone action in the developing brain: Clinical observations and experimental findings J Neuroendocrinol 2004 16 10 809 818 10.1111/j.1365-2826.2004.01243.x 15500540

[B3] Chen ZP Hetzel BS Cretinism revisited Best Pract Res Clin Endocrinol Metab 2010 24 1 39 50 10.1016/j.beem.2009.08.014 20172469

[B4] Skeaff SA Iodine deficiency in pregnancy: the effect on neurodevelopment in the child Nutrients 2011 3 2 265 273 10.3390/nu3020265 22254096 PMC3257674

[B5] Zimmermann MB The role of iodine in human growth and development Semin Cell Dev Biol 2011 22 6 645 652 10.1016/j.semcdb.2011.07.009 21802524

[B6] Teng W Shan Z Teng X Guan H Li Y Teng D Jin Y Yu X Fan C Chong W Yang F Dai H Yu Y Li J Chen Y Zhao D Shi X Hu F Mao J Gu X Yang R Tong Y Wang W Gao T Li C Effects of iodine intake on thyroid diseases in China N Engl J Med 2006 354 26 2783 2793 10.1056/NEJMoa054022 16807415

[B7] Dong J Yin H Liu W Wang P Jiang Y Chen J Congenital iodine deficiency and hypothyroidism impair LTP and decrease c-Fos and c-Jun expression in rat hippocampus Neurotoxicology 2005 26 3 417 426 10.1016/j.neuro.2005.03.003 15935212

[B8] Guzowski JF Insights into immediate-early gene function in hippocampal memory consolidation using antisense oligonucleotide and fluorescent imaging approaches Hippocampus 2002 12 1 86 104 10.1002/hipo.10010 11918292

[B9] Opazo MC Gianini A Pancetti F Azkcona G Alarcón L Lizana R Noches V Gonzalez PA Marassi MP Mora S Rosenthal D Eugenin E Naranjo D Bueno SM Kalergis AM Riedel CA Maternal hypothyroxinemia impairs spatial learning and synaptic nature and function in the offspring Endocrinology 2008 149 10 5097 5106 10.1210/en.2008-0560 18566112 PMC5398426

[B10] Liu D Teng W Shan Z Yu X Gao Y Wang S Fan C Wang H Zhang H The effect of maternal subclinical hypothyroidism during pregnancy on brain development in rat offspring Thyroid 2010 20 8 909 915 10.1089/thy.2009.0036 20615128

[B11] Lasley SM Gilbert ME Developmental thyroid hormone insufficiency reduces expression of brain-derived neurotrophic factor (BDNF) in adults but not in neonates Neurotoxicol Teratol 2011 33 4 464 472 10.1016/j.ntt.2011.04.001 21530650

[B12] Dowling ALS Iannacone EA Zoeller RT Maternal hypothyroidism selectively affects the expression of neuroendocrine-specific protein a messenger ribonucleic acid in the proliferative zone of the fetal rat brain cortex Endocrinology 2001 142 1 390 399 10.1210/en.142.1.390 11145602

[B13] Dong J Liu W Wang Y Hou Y Xi Q Chen J Developmental iodine deficiency resulting in hypothyroidism reduces hippocampal ERK1/2 and CREB in lactational and adolescent rats BMC Neurosci 2009 10 149 10.1186/1471-2202-10-149 20021662 PMC2804698

[B14] Gong J Liu W Dong J Wang Y Xu H Wei W Zhong J Xi Q Chen J Developmental iodine deficiency and hypothyroidism impair neural development in rat hippocampus: involvement of doublecortin and NCAM-180 BMC Neurosci 2010 11 50 10.1186/1471-2202-11-50 20412599 PMC2876162

[B15] Anderson GW Schoonover CM Jones SA Control of Thyroid Hormone Action in the Developing Rat Brain Thyroid 2003 13 11 1039 1056 10.1089/105072503770867219 14651788

[B16] Babikian T Prins ML Cai Y Barkhoudarian G Hartonian I Hovda DA Giza CC Molecular and physiological responses to juvenile traumatic brain injury:focus on growth and metabolism Dev Neurosci 2010 32 5–6 431 441 21071915 10.1159/000320667 PMC3215243

[B17] Evans IM Pickard MR Sinha AK Leonard AJ Sampson DC Ekins RP Influence of maternal hyperthyroidism in the rat on the expression of neuronal and astrocytic cytoskeletal proteins in fetal brain J Endocrinol 2002 175 3 597 604 10.1677/joe.0.1750597 12475371

[B18] Gilbert ME Sui L Walker MJ Anderson W Thomas S Smoller SN Schon JP Phani S Goodman JH Thyroid hormone insufficiency during brain development reduces parvalbumin immunoreactivity and inhibitory function in the hippocampus Endocrinology 2007 148 1 92 102 17008398 10.1210/en.2006-0164

[B19] Hwang S Lee EY Lee WK Shin DY Lee EJ Correlation between iodine intake and thyroid function in subjects with normal thyroid function Biol Trace Elem Res 2011 143 3 1393 1397 10.1007/s12011-011-8997-x 21340678

[B20] Orito Y Oku H Kubota S Amino N Shimogaki K Hata M Manki K Tanaka Y Sugino S Ueta M Kawakita K Nunotani T Tatsumi N Ichihara K Miyauchi A Miyake M Thyroid Function in Early Pregnancy in Japanese Healthy Women: Relation to Urinary Iodine Excretion, Emesis, and Fetal and Child Development J Clin Endocrinol Metab 2009 94 5 1683 1688 10.1210/jc.2008-2111 19258403

[B21] Thomopoulos P Iodine excess and thyroid dysfunction La Revue du Praticien 2005 55 2 180 182 15825999

[B22] Bourdoux PP Ermans AM Mukalay wa Mukalay A Filetti S Vigneri R Iodine induced thyrotoxicosis in Kiwu Zaire Lancet 1996 347 9000 552 553 8596306 10.1016/s0140-6736(96)91188-5

[B23] Fradkin JE Wolff J Iodide-induced thyrotoxicosis Medicine (Baltimore) 1983 62 1 1 20 6218369 10.1097/00005792-198301000-00001

[B24] Connolly RJ Vidor GI Stewart JC Increase in thyrotoxicosis in endemic goitre area after iodation of bread Lancet 1970 1 7645 500 502 4190182 10.1016/s0140-6736(70)91582-5

[B25] Leung AM Braverman LE Iodine-induced thyroid dysfunction Curr Opin Endocrinol Diabetes Obes 2012 19 5 414 419 22820214 10.1097/MED.0b013e3283565bb2 PMC5661998

[B26] Sebotsa ML Dannhauser A Jooste PL Joubert G Iodine status as determined by urinary iodine excretion in Lesotho two years after introducing legislation on universal salt iodization Nutrition 2005 21 1 20 24 10.1016/j.nut.2004.09.005 15661474

[B27] Lewiński A Szybiński Z Bandurska-Stankiewicz E Grzywa M Karwowska A Kinalska I Kowalska A Makarewicz J Nauman J Słowińska-Klencka D Sowiński J Syrenicz A Zonenberg A Huszno B Klencki M Iodine-induced hyperthyroidism–an epidemiological survey several years after institution of iodine prophylaxis in Poland J Endocrinol Invest 2003 26 2 Suppl 57 62 12762642

[B28] Morreale de Escobar G Pastor R Obregon MJ Escobar del Rey F Effects of maternal hypothyroidism on the weight and thyroid hormone content of rat embryonic tissues, before and after onset of fetal thyroid function Endocrinology 1985 117 5 1890 1900 10.1210/endo-117-5-1890 4042969

[B29] Ahmed OM El-Gareib AW El-Bakry AM Abd El-Tawab SM Ahmed RG Thyroid hormones states and brain development interactions Int J Dev Neurosci 2008 26 2 147 209 10.1016/j.ijdevneu.2007.09.011 18031969

[B30] Chen C Zhou Z Zhong M Li M Yang X Zhang Y Wang Y Wei A Qu M Zhang L Xu S Chen S Yu Z Excess thyroid hormone inhibits embryonic neural stem/progenitor cells proliferation andmaintenance through STAT3 signalling pathway Neurotox Res 2011 20 1 15 25 10.1007/s12640-010-9214-y 20711698

[B31] Ahmed OM Abd El-Tawab SM Ahmed RG Effects of experimentally induced maternal hypothyroidism and hyperthyroidism on the development of rat offspring: I. The development of the thyroid hormones-neurotransmitters and adenosinergic system interactions Int J Dev Neurosci 2010 28 6 437 454 10.1016/j.ijdevneu.2010.06.007 20599606

[B32] Emder PJ Jack MM Iodine-induced neonatal hypothyroidism secondary to maternal seaweed consumption: A common practice in some Asian cultures to promote breast milk supply J Paediatr Child Health 2011 47 10 750 752 10.1111/j.1440-1754.2010.01972.x 21276114

[B33] Gao TS Teng WP Shan ZY Jin Y Guan HX Teng XC Yang F Wang WB Shi XG Tong YJ Li D Chen W Effect of different iodine intake on schoolchildren thyroid diseases and intelligence in rural areas Chin Med J (Engl) 2004 117 10 1518 1522 15498376

[B34] Chung HR Shin CH Yang SW Choi CW Kim BI Subclinical hypothyroidism in Korean preterm infants associated with high levels of iodine in breast milk J Clin Endocrinol Metab 2009 94 11 4444 4447 10.1210/jc.2009-0632 19808851

[B35] Herdegen T Skene P Bähr M The c-Jun transcription factor—bipotential mediator of neuronal death, survival and regeneration Trends Neurosci 1997 20 5 227 231 10.1016/S0166-2236(96)01000-4 9141200

[B36] Wang Y Su B Xia Z Brain-derived neurotrophic factor activates ERK5 in cortical neurons via a Rap1-MEKK2 signaling cascade J Biol Chem 2006 281 47 35965 35974 10.1074/jbc.M605503200 17003042

[B37] Lewin GR Barde YA Physiology of the neurotrophins Annu Rev Neurosci 1996 19 289 317 10.1146/annurev.ne.19.030196.001445 8833445

[B38] Lu B Figurov A Role of neurotrophins in synapse development and plasticity Rev Neurosci 1997 8 1 1 12 9402641 10.1515/revneuro.1997.8.1.1

[B39] Heldt SA Stanek L Chhatwal JP Ressler KJ Hippocampus-specific deletion of BDNF in adult mice impairs spatial memory and extinction of aversive memories Mol Psychiatry 2007 12 7 656 670 10.1038/sj.mp.4001957 17264839 PMC2442923

[B40] Chakraborty G Magagna-Poveda A Parratt C Umans JG MacLusky NJ Scharfman HE Reduced hippocampal brain-derived neurotrophic factor (BDNF) in neonatal rats after prenatal exposure to propylthiouracil (PTU) Endocrinology 2012 153 3 1311 1316 10.1210/en.2011-1437 22253429 PMC3384077

[B41] Wang S Teng W Gao Y Fan C Zhang H Shan Z Early levothyroxine treatment on maternal subclinical hypothyroidism improves spatial learning of offspring in rats J Neuroendocrinol 2012 24 5 841 848 10.1111/j.1365-2826.2011.02275.x 22192600

[B42] Senden NH Timmer ED Boers JE van de Velde HJ Roebroek AJ Van de Ven WJ Broers JL Ramaekers FC Neuroendocrine-specific protein C (NSP-C): subcellular localization and differential expression in relation to NSP-A Eur J Cell Biol 1996 69 3 197 213 8900485

[B43] Dowling ALS Martz GU Leonard JL Zoeller RT Acute changes in maternal thyroid hormone induce rapid and transient changes in gene expression in fetal rat brain J Neurosci 2000 20 6 2255 2265 10704501 10.1523/JNEUROSCI.20-06-02255.2000 PMC6772490

[B44] Brown RW Flanigan TJ Thompson KN Thacker SK Schaefer TL Williams MT Neonatal quinpirole treatment impairs Morris water task performance in early postweanling rats: relationship to increases in corticosterone and decreases in neurotrophic factors Biol Psychiatry 2004 56 3 161 168 10.1016/j.biopsych.2004.05.003 15271584

